# Tobacco use patterns, knowledge, attitudes towards tobacco and availability of tobacco control training among school personnel from a rural area in Poland

**DOI:** 10.1186/s12971-016-0110-y

**Published:** 2017-01-11

**Authors:** Dorota Kaleta, Kinga Polańska, Adam Rzeźnicki, Włodzimierz Stelmach, Piotr Wojtysiak

**Affiliations:** 1 Department of Tobacco Control, Preventive Medicine Department, Medical University of Lodz, Zeligowskiego 7/9 Str, 90-752 Lodz, Poland; 2 Department of Social Medicine, Medical University of Lodz, Zeligowskiego 7/9 Str, 90-752 Lodz, Poland

**Keywords:** School personnel, Smoking, Tobacco control training, Health policy

## Abstract

**Background:**

Tobacco-free school environment as well as non-smoking teachers and school personnel provide positive role models for children and young people. In Poland, smoking should be banned in colleges, schools, educational establishments and educational care facilities. However, for the existing law to be effective, awareness of all people in school curriculum and enforcement of the law are crucial. The aim of the study was to evaluate tobacco use patterns, knowledge and attitudes towards tobacco as well as availability of tobacco control training among school personnel in a rural area in Poland. Moreover, compliance with tobacco control policies and their enforcement were assessed.

**Methods:**

The study was carried out in Piotrkowski district between November 2014 and May 2015 in accordance with the Global School Personnel Survey (GSPS) methodology. Sixty schools participated in the survey (92% of the schools from the region) with involvement of 1044 teachers and 500 non-teaching staff (the response rate – 83.1%). The multivariate linear regression analyses were applied to study factors linked to the need for anti-tobacco training dedicated to the youth and teachers’ knowledge as well as activities to educate the students about tobacco use and its prevention.

**Results:**

About 24% of the school personnel were current and 9% were ex-smokers. Significantly more teachers than the non-teaching staff indicated that the schools had a policy prohibiting tobacco use among students. In addition, 6% of the study participants indicated everyday violations of the tobacco control policy by the school personnel. More than 80% of the teaching personnel indicated the need for training dedicated to the youth to prevent their tobacco use. In the multivariate linear regression model, longer duration of working experience predicted higher levels of knowledge and more activities performed to teach the youth about tobacco use and its prevention. The smokers comparing to the non–smokers perceived the need for anti- tobacco training among the youth less strongly.

**Conclusions:**

In order to make it possible for the inhabitants of Piotrkowski district to work and learn in tobacco smoke free environment there is an urgent need for taking actions aiming at increasing effectiveness of enforcing applicable tobacco control regulations in educational units. The necessity for systematic training dedicated to the youth to prevent their tobacco use, including accurate preparation of teachers, also needs to be highlighted.

**Electronic supplementary material:**

The online version of this article (doi:10.1186/s12971-016-0110-y) contains supplementary material, which is available to authorized users.

## Background

Health effects of smoking are well established. It is estimated that half of those who smoke and fail to stop die because of their habit [1, 2]. Despite that, most smokers start smoking before they reach the age of 18 years [3]. Data from the Global Adult Tobacco Survey (GATS) have indicated that in Poland among people older than 15 years of age, about 33% of men and 21% of women smoked cigarettes on a daily basis, and more than 3% of them smoked occasionally [4–8]. The high prevalence of smoking is also observed among the youth. Based on the Health Behavior in School-aged Children (HBSC) study among 11–12 year olds, about 16% of boys and 9% of girls had already tried their first cigarette. Among older youths (15–16 years old) 10% of pupils indicated at least one cigarette per day (including 12% of boys and 8% of girls) [9]. A variety of factors are indicated as predictors of the onset of adolescent smoking, including: socio-demographic influences (socio-economic status, gender, age, availability of money to spend), social bonding factors (family and peer bonding, school influences), social learning factors (family and peer smoking) and personal characteristics (low self-esteem, refusal skills, beliefs and attitudes towards tobacco including the perception that tobacco use is a norm) [3, 10–13].

One of the most important strategies in reducing smoking prevalence in the population is to prevent young people from becoming smokers [3, 14, 15]. School-based strategies are one of the key elements of adolescent tobacco control because school environments are established systems in which adolescent behavior can be targeted and in which social behaviors are reinforced [10, 11, 16–23]. Teachers and school personnel have a central role in shaping young people’s attitude towards smoking [16]. Their daily interactions and strong influence on students potentially make them an influential group for tobacco control [24]. Teacher’s knowledge, behavior and attitudes towards smoking education are closely related to their own smoking status [16, 25]. Studies indicate that never and ex-smoker teachers have the most positive role in preventing smoking among the youth [16, 21, 25–30]. On the contrary, those who smoke may influence adolescents to adopt smoking through direct modeling [25–30]. Schools with a higher percentage of smokers among school staff members or among senior students may increase the risk of smoking among their students. Previous studies have shown that schools with anti-smoking policies have a reduced probability of student exposure to teachers who smoke in school as well as significantly lower prevalence of student smoking [25–30].

Existence, awareness and enforcement of the legislation banning smoking in a school environment seems to be a significant element for the prevention of tobacco use among young people. However, it needs to go in parallel with educational activities, which can be performed by teachers [14]. A teacher, apart from the obligation to teach a particular subject should be a role model and be responsible for upbringing and public health issues. In order to educate students in the field of tobacco control, teachers need to have relevant knowledge and skills, therefore, training to prepare them for such activities is crucial [3, 10, 11, 16].

In Poland, based on the amendment of the Act on Protection of Health against Consequences of Consumption of Tobacco and Tobacco Products and the Act on National Sanitary Inspectorate (of 8 April 2010), smoking should be banned in colleges, schools, educational establishments, educational care facilities and in foster care centres mentioned in the regulations on social assistance [31]. In addition, an owner or manager of a facility where smoking is prohibited should visibly display adequate written information and graphic symbols advising on the smoking ban on the premises. The existing legislation also emphasizes the fact that it is a duty of a country to protect people from health effect of tobacco use by formulation of health, economic and social policies. Such policies should aim at protection of the right of non-smokers to live in a tobacco smoke-free environment, health promotion through dissemination of a free from smoking, healthy lifestyle as well as educational and information activities raising public awareness of the dangers of tobacco consumption.

Other important point that needs to be strengthened is high influence of tobacco industry on young people. Despite the existence of legislation in Poland that regulates and minimizes activities of tobacco companies, they use a diversity of tactics to obtain new consumers [32, 33]. The most common practices represented by tobacco industry are: creating a positive image, which allows concluding cooperation with various media (including social media), as well as direct and indirect advertising. Brand stretching, product advertising, supporting public events, trade fairs, recruitment strategies as well as hiring, awards, and rankings are activities commonly observed in Poland [32, 33]. Such practices influence young people’s knowledge, attitudes and perception of tobacco. In that respect, appropriate education, which can be conducted by teachers in a school curriculum, can be crucial to help young people to make informed decisions about their smoking status.

It needs to be pointed that although in Poland national surveillance of tobacco smoking, enforcement of the law and training activities dedicated to tobacco control are pretty well elaborated, they are mostly performed in big cities. At the very same time smaller cities/towns are poorly covered by such initiatives [34–38].

Taking this into account, the aim of the study was to evaluate tobacco use patterns, knowledge and attitudes towards tobacco as well as availability of tobacco control training among school personnel in a rural area in Poland. Moreover, compliance with tobacco control policies and their enforcement in schools from such an area were assessed.

## Methods

### Study design and population

The cross-sectional study was carried out in Piotrkowski district between November 2014 and May 2015 in accordance with the Global School Personnel Survey (GSPS) methodology. GSPS is a part of Global Tobacco Surveillance System (GTSS) developed by the World Health Organization (WHO), Centers for Disease Control (CDC) and Prevention and Canadian Public Health Association [16, 39]. This school based survey of school personnel (teachers and non-teaching staff) uses a standard methodology and core questionnaire to systematically monitor school personnel tobacco use, their knowledge and attitudes toward tobacco. Moreover, it evaluates existence and effectiveness of tobacco control policies in schools, existence of training materials on tobacco prevention as well as existence of curriculum on tobacco prevention, and control interventions.

Potrkowski district is a socially disadvantaged rural area in central Poland (Lodzkie voivodeship – an administrative region of central Poland). According to the state as of 2013, more than 90% of the residents of the district (82,854 individuals) were people who lived in the rural area. In 2012, 23% of its residents of the district required support of social assistance institutions due to the lack of resources to live on [40]. It also needs to be pointed that unemployment rate in the district is higher than the national unemployment rate. Simultaneously, it has to be emphasized that the rate of unemployment, in the context of the rural nature of the district, is underestimated as a social problem. The analysis performed by the United Nations Development Programme (UNDP), placed Piotrkowski district at the 11th position among 30 districts of all 314 rural districts that exist in Poland, with the lowest indicators of social development. Local Human Development Index (LHDI) covering three indicators: Health Index, Education Index, Welfare Index was 25.97, including Health Index HI = 26.50, whereas the discussed indicators for Lodzkie voivodeship were, respectively: 39.28 and 31.48 [41].

All 65 schools without any exclusions from Piotrkowski district were invited to participate the study. The sixty schools (including primary schools with students aged 7–12 years, secondary schools with students aged 13–15 years and high schools with students aged 16–19 years) participated in the survey (92% of the schools from this region) with involvement of 1544 school personnel including 1044 teachers and 500 non-teaching staff (the response rate – 83.1%). There were 13 659 total number of students in these schools. A written informed consent was obtained from all of the study participants.

The study obtained a positive opinion from the Bioethics Committee of Medical University in Lodz [decision number: RNN/730/14/KB].

### Questionnaire

An anonymous, self-administered questionnaire was filled in by all the study participants. The core questionnaire consisted of 45 questions divided into 5 categories: tobacco use, school policy, attitudes towards tobacco control issues, training and demographic data. Additional 48 questions covered policy issues in Poland (legislation dedicated to a school curriculum, its effectiveness and enforcement of the law), environmental tobacco smoke exposure at home and in public places, and knowledge on consequences of smoking and ETS exposure. The respondents were asked if their school had a policy banning smoking in a school building, school area and during school events (policy for school personnel and the youth), violation of tobacco control policy, visible information about prohibiting smoking in the school building and school area. Teachers were asked about their responsibility, knowledge and practical skills in providing anti-smoking education and intervention among pupils. Socio-demographic data covered: gender, age, marital status, income and duration of working experience.

Current smokers were defined as the people who smoked cigarettes on a daily basis and occasionally (less than one cigarette per day), ex-smokers as those who had ever (but not currently) smoked cigarettes.

The teacher’s perception of the need for training dedicated to the youth to prevent their tobacco use (indicator 1) was assessed based on their agreement with 3 statements - namely: 1) “Information about health consequences of smoking given by professionals has impact on the youth’s decision about tobacco use (initiation or cessation of smoking)”; 2) “There is a need for training dedicated to the youth to prevent their tobacco use”; 3) “Parents expect an in-school training for the youth to prevent their tobacco use”. For each of the statements there were answers, which were converted in the analysis into the points: 3 = yes, 2 = I do not know and 1 = no (with potential maximum – 9 points and potential minimum – 3 points; the higher number of points, the higher the perceived need for training to prevent tobacco use). The second indicator (indicator 2) was calculated to describe teachers’ knowledge and activities performed to educate the youth about tobacco use and its prevention. This was assessed based on their agreement with 3 separate statements: 1) “I have knowledge necessary to educate how to prevent the youth tobacco use”, 2) “I taught the youth how to avoid tobacco use” and 3) “I conduct non-classroom activities to teach about tobacco use and its prevention” with scoring given for each statement from 1 = no to 2 = yes (with potential maximum – 6 points and potential minimum – 3 points; the higher number of points, the higher the teacher’s knowledge and greater number of anti-smoking activities performed). Finally, the points were used as continuous values to estimate the mean ranking for each indicator.

### Statistical analysis

The data were entered into the Excel data analysis software on a daily basis by field investigators, and then submitted to a supervisor. Once the data collection process were completed, 5% of the records were randomly checked to confirm that they were clearly recorded, complete and consistent across responses. The dataset supporting the conclusions of this article is included within the article as an additional file (Additional file 1). Data analysis was performed using STATISTICA 10.0.

Data are presented as numbers and percentages. Ninety-five percent confidence intervals (CI 95%) and *p* value were calculated and used to test for significance of difference. The Chi-squared test for independence and large sample test for proportions were applied.

The results are presented as overall mean scores (± SD) on indicator 1 and 2 and scores presented for gender, duration of working experience and a teacher’s smoking status. Significance of differences between the subgroups was tested using the independent sample *t* test. Finally, the association between selected variables (age, duration of working experience, smoking status) and scores of indicator 1 and 2 was tested by the linear regression model with adjustment for confounding from the other factors.

## Results

### Socio-demographic characteristic and tobacco smoking among the study population

Socio demographic characteristics and smoking status of the study participants are presented in Table 1. Of the total of 1544 respondents, 86% were females and half indicated over 10 years of working experience. More than 80% of the study participants were married and nearly 70% were allocated into a low income category based on monthly net income for one person in the household (<1500PLN).

**Table 1:** Prevalence of tobacco smoking among teachers (*N* = 1044) and non-teaching staff (*N* = 500) in Piotrkowski district

Characteristics	Teachers			Non-teaching staff		
	Current smoker *N* = 221 (21.2%)	Ex-smoker *N* = 69 (6.6%)	Never smoker *N* = 754 (72.2%)	Current smoker *N* = 149 (29.8%)	Ex-smoker *N* = 64 (12.8%)	Never smoker *N* = 287 (57.4%)
	n (%) 95% CI			n (%) 95% CI		
Gender						
Male (*N* = 223)	33 (25.0)	13 (9.9)	86 (65.2)	28 (30.8)	24 (26.4)^a^	39 (42.9)^a^
	17.6–32.4	4.8–15.0	57.1–73.3	21.3–40.3	17.3–35.5	32.7–53.1
Female (*N* = 1321)	188 (20.6)	56 (6.1)	668 (73.2)	121 (29.6)	40 (9.8)	248 (60.4)
	13.7–27.5	2.0–10.2	65.6–80.8	25.2–34.0	6.9–12.7	55.7–65.1
Age range (years)						
< 35 (*N* = 285)	36 (16.3)^b^	12 (5.4)	173 (78.3)	14 (21.9)	1 (1.6)^b,c^	49 (76.6)^b^
	11.4–21.2	2.4–8.4	72.9–83.7	11.8–32.0	0.1–4.7	66.2–87.0
35–44 (*N* = 498)	66 (18.2)	15 (4.1)^d^	281 (77.6)^d^	28 (20.6)^d^	22 (16.2)	86 (63.4)
	14.2–22.2	2.1–6.1	73.3–81.9	13.8–27.4	10.0–22.4	55.3–71.5
> 44 (*N* = 761)	119 (25.8)	42 (9.1)	300 (65.1)	107 (35.7)	41 (13.7)	152 (50.7)
	21.8–29.8	6.5–11.7	60.7–69.4	30.3–41.2	9.8–17.6	45.0–56.4
Duration of working experience						
< 2 years (*N* = 195)	18 (16.7)	8 (7.4)	82 (75.9)	18 (20.7)^e^	8 (9.2)	61 (70.1)^e^
	9.7–23.7	2.5–12.3	67.8–84.0	12.2–29.2	3.1–15.3	60.5–79.7
2 years to 10 years (*N* = 542)	75 (21.3)	19 (6.0)	262 (72.8)	51 (27.4)	29 (15.6)	106 (57.0)
	17.1–25.5	3.5–8.5	68.2–77.4	21.0–33.8	10.4–20.8	49.9–64.1
over 10 years (*N* = 807)	128 (22.1)	42 (7.2)	410 (70.7)	80 (35.2)	27 (11.9)	120 (52.9)
	18.7–25.5	5.1–9.3	67.0–74.4	29.6–40.8	8.1–15.7	47.0–58.8
Marital status						
Unmarried^#^ (*N* = 279)	48 (26.2)	8 (4.4)	127 (69.4)	33 (34.4)	17 (17.7)	46 (47.9)
	19.8–32.6	1.4–7.4	62.7–76.1	24.9–43.9	10.1–25.3	37.9–57.9
Married (*N* = 1265)	173 (20.1)	61 (7.1)	627 (72.8)	116 (28.7)	47 (11.6)	241 (59.7)
	17.4–22.8	7.2–11.0	69.8–75.8	24.3–33.1	8.5–14.7	54.9–64.5
Income*						
High >2500 PLN (*N* = 94)	21 (29.6)	8 (11.3)	42 (59.1)^f^	4 (17.4)	4 (17.4)	15 (65.2)
	19.0–40.2	3.9–18.7	47.8–70.6	1.9–32.9	1.9–32.9	45.7–84.7
Medium (1500–2500 PLN) (*N* = 415)	73 (22.5)	22 (6.8)	229 (70.7)	30 (33.0)	10 (11.0)	51 (56.0)
	18.0–27.0	4.1–9.5	65.7–75.7	23.3–42.7	4.6–17.4	45.8–66.2
Low (<1500 PLN) (*N* = 1035)	127 (19.6)	39 (6.0)	483 (74.4)	115 (29.8)	50 (13.0)	221 (57.2)
	16.6–22.7	4.2–7.8	71.0–77.8	25.4–34.4	9.6–16.4	52.3–62.1

^a^male vs. female *p* < 0.002; ^b^age <35 vs. >44 *p* < 0.02; ^c^age <35 vs. 35–44 *p* < 0.005; ^d^age 35–44 vs. >44 *p* < 0.006; ^e^duration of working < 2 years vs over 10 years *p* < 0.01; ^f^high vs low income *p* < 0.006

^#^Unmarried – combined categories: single, divorced, widowed

*Income based on the question “What is the monthly net income for one person in household?

Among the participants, 24% indicated that they were currently smoking cigarettes and 9% were ex-smokers (Table 1). The percentages of the current and ex-smokers were higher among the non-teaching than teaching staff (current smokers: 30% vs. 21% and ex-smokers: 13% vs. 7%). In the group of the non-teaching staff there were significantly more never-smokers among females than males. Smoking status also differed depending on the age of the respondents with higher proportion of smokers being observed among the older participants. Among the teachers with higher income, never-smoking status was indicated significantly less frequently than in the group with less money available per one person in the household (low income category).

### School policies prohibiting tobacco use

Table 2 presents knowledge of school personnel concerning existence of tobacco control policies in their school. Significantly more teachers than the non-teaching staff indicated that the schools had a policy prohibiting tobacco use among students (*p* < 0.001). It is important that the percentage of school personnel who indicated existence of the policy prohibiting smoking by the school staff was lower when compared to that focused on students More than 60% of the school personnel indicated enforcement of a school policy and about 6% of them indicated everyday violations of the tobacco control policy. Significantly fewer teachers (79%) than the non-teaching staff (91%) indicated that their school had visible information about prohibiting smoking in the school and on its premises (*p* < 0.001).

**Table 2:** Existing tobacco control policies and practices

	Teachers *N* = 1044		Non-teaching staff *N* = 500		*p*-value*
	N (%)	(CI 95%)	N (%)	(CI 95%)	
Policy prohibiting tobacco use among students					
In school building	782(74.9)	72.3–77.5	294(58.8)	54.5–63.1	<.001
School area	756(72.4)	69.7–75.1	283(56.6)	52.3–60.9	<.001
On school events	731(70.0)	67.2–72.8	278(55.6)	51.2–60.0	<.001
Policy prohibiting tobacco use among school personnel					
In school building	505(48.4)	45.4–51.4	230(46.0)	41.6–50.4	
School area	452(43.3)	40.3–46.3	212(42.2)	38.1–46.7	
On school events	456(43.7)	40.7–46.7	220(44.0)	39.7–48.4	
Enforcement of a school policy					
Yes	490(66.0)	62.6–69.4	229(67.8)	62.8–72.8	
No	83(11.2)	8.9–13.5	28(8.3)	5.4–11.2	
I do not know	169(22.8)	19.8–25.8	81(24.0)	19.5–28.6	
Violation of the tobacco control policy by school personnel					
Everyday	60(5.8)	4.4–7.2	30(6.0)	3.9–8.1	
Once/week or once/month or less than one/month	36(3.4)	2.3–4.5	27(5.4)	3.4–7.4	
Never	443(42.4)	39.4–45.5	185(37.0)	32.8–41.2	
I do not know	505(48.4)	45.5–51.4	258(51.6)	47.2–56.0	
Visible information about prohibiting smoking in the school and on its premises					
Yes	820(78.5)	76.0–81.0	453(90.6)	88.0–93.2	<.001
No	123(11.8)	9.8–13.8	28(5.6)	3.6–7.6	<.001
I do not know	101(9.7)	7.9–11.5	19(3.8)	2.1–5.5	

*p value is indicating differences between teachers and non-teaching staff

### Knowledge, attitudes towards tobacco

There are significant differences between the teachers and non-teaching staff in terms of knowledge and the opinion on tobacco control issues (Table 3). More than 90% of the teachers and 83% of the non-teaching personnel agreed with the statement that active and passive smoking causes serious diseases (*p* < 0.001). Similarly, significantly more teachers comparing to other school personnel shared the opinion that smoking should be banned in all workplaces and governmental offices (*p* ≤ 0.006) as well as that tobacco advertising should be completely banned (*p* < 0.001).

**Table 3:** Opinion on tobacco control issues

	Teachers 1044		Non-teaching staff 500		*p*-value*
	N(%)	(CI 95%)	N(%)	(CI 95%)	
Concerns over health consequences of smoking**					
Yes	210(89.7)	85.8–93.3	132(84.1)	78.4–89.8	
No	24(10.3)	6.4–14.2	25(15.9)	11.2–21.6	
Tobacco causes serious diseases in smokers					
Disagree	37(3.5)	2.4–4.2	29(5.8)	4.3–7.8	.040
I do not have opinion	39(3.7)	2.6–4.8	55(11.0)	8.3–13.7	<.001
Agree	968(92.8)	91.2–94.4	416(83.2)	79.9–86.5	<.001
ETS causes serious diseases in non-smokers					
Disagree	48(4.6)	3.3–5.9	24(4.8)	2.9–6.7	
I do not have opinion	47(4.5)	3.2–5.8	61(12.2)	9.3–15.1	<.001
Agree	949(90.9)	89.2–92.6	415(83.0)	79.7–86.3	<.001
Smoking should be banned in all workplaces					
Yes	946(90.6)	88.8–92.4	430(86.0)	82.9–88.7	.006
No	47(4.5)	3.2–5.3	38(7.6)	5.4–9.9	.012
I do not know	51(4.9)	3.6–6.3	32(6.4)	4.2–8.6	
Smoking should be banned in all governmental offices					
Yes	979(93.8)	92.3–95.3	447(89.4)	86.7–92.1	.003
No	25(2.4)	1.5–3.3	28(5.6)	3.6–7.6	.001
I do not know	40(3.8)	2.6–5.0	25(5.0)	3.1–6.9	
Prices of tobacco products should be increased					
Disagree	329(31.5)	28.7–34.3	177(35.4)	31.2–39.6	
I do not have opinion	307(29.4)	26.6–32.2	154(30.8)	26.8–34.8	
Agree	408(39.1)	36.1–42.1	179(35.8)	31.6–40.0	
Tobacco advertising should be completely banned					
Disagree	114(10.9)	9.1–12.8	58(11.8)	9.0–14.6	
I do not have opinion	177(17.0)	14.7–19.3	139(27.8)	23.9–31.7	.001
Agree	753(72.1)	69.4–74.8	303(60.6)	56.3–64.9	<.001
Pictorial warnings should be introduced on tobacco packages					
Disagree	77(7.3)	5.7–8.6	52(10.4)	8.7–13.1	.044
I do not have opinion	216(20.7)	18.2–23.2	112(22.4)	18.8–26.1	
Agree	751(72.0)	69.3–74.7	336(67.2)	63.1–71.3	

*p value is indicating differences between teachers and non-teaching staff

**Question dedicated only to smokers: *N* = 234 among the teachers. *N* = 157 among the non-teaching staff

### Training related curriculum

Descriptive data about training and access to teaching materials are presented in additional file (Additional file 2: Table S1). More than 80% of the teaching personnel indicated the need for training dedicated to the youth in order to prevent their tobacco use. Tables 4 and 5 presents the mean scores for the need for training dedicated to the youth to prevent their tobacco use (indicator 1) and knowledge as well as activities performed to teach the youth about tobacco use and its prevention (indicator 2). There are no significant differences in the mean scoring of that indicators regarding gender status of the respondents. Interestingly, the teachers with longer duration of working experience more strongly perceived that there is a need for anti-tobacco training among the youth than those who had a shorter working period (*p* = 0.05). They also received higher scoring for knowledge they had and anti-tobacco activities performed among the students (*p* = 0.003). The smokers had a lower score of indicator describing the need of the training than that obtained in the group of non-smokers.

**Table 4:** Mean scores for the need for training dedicated to the youth to prevent their tobacco use (indicator 1) and knowledge and performed activities to teach the youth about tobacco use and its prevention (indicator 2) by teacher’s gender, duration of working experience and smoking status

Indicators	Total *N* = 1044	Male *N* = 132	Female *N* = 912	t (*p* value)	Working experience <10 years *N* = 464	Working experience ≥10 years *N* = 579	t (*p* value)	Current smoker *N* = 221	Non-smoker *N* = 823	t (*p* value)
	Mean ± SD									
Indicator 1	7.77 ± 1.29	7.58 ± 1.45	7.79 ± 1.26	.15	7.71 ± 1.25	7.81 ± 1.32	.05	7.62 ± 1.33	7.80 ± 1.27	.059
Indicator 2	5.21 ± 0.74	5.22 ± 0.68	5.21 ± 0.75	.87	5.13 ± 0.77	5.28 ± 0.71	.003	5.27 ± 0.72	5.20 ± 0.74	.21

Indicator 1 - need for training dedicated to the youth to prevent their tobacco use based on 3 statements and scoring given for each statement from 1 = no, 2 = I do not know and 3 = yes (potential maximum 9 points; potential minimum 3 points) - the points were used as continuous variables to estimate the mean ranking for indicator

Indicator 2 - knowledge and performed activities to teach the youth about tobacco use and its prevention (based on 3 statements and scoring given for each statement from 1 = no to 2 = yes (potential maximum 6 points; potential minimum 3 points) - the points were used as continuous variables to estimate the mean ranking for indicator

±SD standard deviation

**Table 5:** Associations between perceived need for training dedicated to the youth to prevent their tobacco use (indicator 1) and knowledge and performed activities to teach the youth about tobacco use and its prevention (indicator 2) and age, duration of working experience and smoking status

Indicators	Gender (male vs. female)	Duration of working experience (continuous variable)	Current smoking status (yes vs. no)
	β(*p* value)		
Indicator 1	−0.049 (.111)	0.028 (.366)	−0.056 (.070)
Indicator 2	0.010 (.742)	0.082 (.008)	0.036 (.246)

Indicator 1 - need for training dedicated to the youth to prevent their tobacco use based on 3 statements and scoring given for each statement from 1 = no, 2 = I do not know and 3 = yes (potential maximum 9 points; potential minimum 3 points) - the points were used as continuous variables to estimate the mean ranking for indicator

Indicator 2 - knowledge and performed activities to teach the youth about tobacco use and its prevention (based on 3 statements and scoring given for each statement from 1 = no to 2 = yes (potential maximum 6 points; potential minimum 3 points) - the points were used as continuous variables to estimate the mean ranking for indicator

β regression coefficients presented for each variable reflect their association with indicator 1 and 2 after adjustment for confounding from the other factors

In the multivariate linear regression model, a longer working experience predicted higher levels of knowledge and greater number of activities performed to teach the youth about tobacco use and its prevention (*p* = 0.008) (Table 5). The smokers comparing to the non–smokers less strongly perceived the need for anti-tobacco training among the youth (*p* = 0.07).

## Discussion

The study results indicate that about quarter of the school personnel currently smoke cigarettes. Significantly more teachers than the non-teaching staff indicated that their schools had a policy prohibiting tobacco use among students. High proportion of the teaching personnel declared the need for training dedicated to the youth to prevent their tobacco use. In addition longer duration of working experience predicted higher levels of knowledge and more activities performed to teach the youth about tobacco use and its prevention. The smokers comparing to the non–smokers perceived the need for anti-tobacco training among the youth less strongly.

School personnel can play an important role in a tobacco control because of their status as role models in communities and frequent contacts with the youth [10, 11, 16–23]. It needs to be pointed that such a potential can be limited by the personnel’s smoking status [16, 21, 25–30]. The smoking prevalence observed in the current analysis is comparable to that noticed in GATS conducted in Poland between the years 2009 and 2010 (27% of daily smokers) and higher than the average, which was reported in the global report of GSPS (15–19%) [4–7, 16]. Compared to the results of our study, the data from GSPS conducted in the European region have indicated similar percentages of current smokers in Slovakia (24%) and Slovenia (22%), higher in Bulgaria (48%) but lower in the Czech Republic (20%) [16].

In addition to reducing exposure of the youth and school personnel to environmental tobacco smoke, the strength and enforcement of a school policy prohibiting smoking are associated with a lower level of tobacco consumption and its prevalence among pupils [14, 16, 25–30]. Despite existing legislation banning smoking in school building, school area and on school events, in or study only 70% of the school personnel indicated existence of a policy prohibiting tobacco use among students and even fewer of a policy prohibiting tobacco use among the school personnel (less than 50%). The GSPS conducted in other European countries indicated that a higher proportion of school staff indicated that a school had a policy prohibiting tobacco use among students in Slovakia (84%) and lower in Slovenia (63%) [16]. Similarly as in our study, in other countries such percentages were lower for regulations dedicated to the staff. It is important to be aware that GSPS in other countries was conducted earlier than our study so they cannot be directly compared as legislation could have improved over time.

The policy needs to be applied to all indoor and outdoor areas of the school. Seeing an adult smoking, including the outdoor areas, increases the likelihood of smoking by the youth and undermines educational messages and other prevention efforts to reduce adolescent smoking [42–44]. In our study, percentage of the school personnel indicating prohibiting of smoking in school areas or during school events is slightly lower that that dedicated to school building, which strengthens the importance of education and awareness of existing legislation.

Several empirical studies suggest that the major factors predicting onset of smoking are socio-environmental factors, including exposure to smoker, role models in the family, peer and school settings, and the perception that tobacco use is the norm. School setting represents also one of the crucial arenas in which learning takes place. The school has long been considered as an important setting for child development and health behaviors, and many smoking prevention programs are school based. Moreover, exposure to teachers and non-teaching staff smoking during school hours may influence smoking behavior of adolescents. School staff who smoke may, therefore, influence adolescents to adopt smoking through direct modelling.

Previous studies have shown that schools with antismoking policies have a reduced probability of student exposure to teachers who smoke in school as well as significantly lower prevalence of student smoking [21, 26–28].

The small number of studies that have looked at the effects of school smoking policy on adolescent smoking behavior suggest that the prevalence of smoking declines when there is a ban on student smoking on the school grounds [45, 46]. According to the study of Poulsen et al., adolescents’ perceived exposure to teachers smoking outdoors on the school premises was significantly associated with daily smoking, having adjusted for sex, exposure to teachers smoking indoors at school and pupils smoking outdoors at school, as well as the smoking behavior of a mother, father, and best friend (odds ratio (OR) 1.8, 95% confidence interval 1.2 to 2.8) [10]. Teachers and administrators are the role models for students, conveyors of tobacco prevention curricula and key opinion leaders for school tobacco control policies. The majority of tobacco users first try tobacco in their teens, and school is mandatory in most countries through age 15 or 16. School teachers and administrators have daily interaction with students and thus, represent an influential group for tobacco control [16, 47, 48].

The other issue, which needs to be pointed, is enforcement of the existing legislation. In our analysis, about 65% of the participants indicated that their school enforced its tobacco policy and 6% indicated its everyday violations by school personnel. Higher percentages in the respect of the enforcement of the legislation were observed in the Czech Republic (76%) and Slovakia (75%), but lower in Slovenia (45%) [16]. Youth smoking prevention and control efforts have had mixed results. However, this review suggests a number of prevention strategies that are promising, especially if conducted in a coordinated way to take advantage of potential synergies across interventions. Several types of strategies warrant additional attention and evaluation, including aggressive media campaigns, teen smoking cessation programmes, social environment changes, community interventions, and increasing cigarette prices [49].

School programs are often one of the most important approaches mentioned in efforts to denormalize tobacco [14]. Willingness to conduct anti-smoking activities among the youth, access to appropriate educational materials and knowledge are important elements of an effective curriculum to prevent and reduce tobacco use among students [14, 50–52]. Early training might help students resist temptations from peers to smoke [14]. The Cochrane review of the school-based programmes for preventing smoking indicated that Pure Prevention cohorts showed a significant effect at longest follow-up, with an average 12% reduction in starting smoking compared to the control groups. The combined social competence and social influences interventions showed a significant effect at one year and at longest follow-up [53]. Most of our study participants expressed the need for a training dedicated to the youth to prevent their tobacco use, but only about half of them thought that information about health consequences of smoking given by a professional had impact on the youth’s decision about tobacco use. In addition, one third declared not enough knowledge to conduct such activities. This pointed the need to pay more attention to the effective preparation of teachers so that they would be able and ready to provide prevention activities. For effective school-based programs they need to follow the standards necessary for quality comprehensive education [14]. The USA National Health Education Standards require that students: (a) comprehend the health risks, (b) analyse the influences of family, peers, culture and media on usage patterns, (c) develop interpersonal skills to resist temptations and (d) practice goal setting and decision making skills to protect against use [54]. While importance of a further training of the school personnel and the youth about specific health hazards of tobacco use needs to be pointed, it is not likely that such a training alone will be sufficient to assure that tobacco use prevention is incorporated into school curricula [14, 24]. Tobacco use prevention and its reduction among the youth requires a comprehensive approach involving teachers, non-teaching staff, parents and other influential persons. Support cessation for teachers, staff and students need to be also pointed [14]. More training may enhance their knowledge towards smoking hazards, better estimation of the problem and planning appropriate interventions to reduce smoking and enforce smoking policies at schools. In our study, most of the teachers expressed their willingness to contribute to that. However, the teachers’ ability to convey effective anti-smoking activities among the youth can be diminished if they have not received a specific training in that field or do not have access to adequate training materials to support anti-tobacco curricula. Research of other authors indicates that the vast majority of teachers believe that an additional training for them in the field of tobacco control activities among young people is necessary. They feel convinced they should undergo such an additional training in order to increase their skills and thus, to be able to prevent young people from smoking initiation or continuation effectively [55–57].

Non-teaching staff have lesser contact with school children than the teachers but still, every day they actively participate in the school life (e.g., librarian, secretary services or cleaning activities etc.). Such people have contact with children, are seen by them during performing their professional activities, during breaks and similarly to the teachers they have to be a role models. Moreover, they are adults themselves so they have to obey the law and anti-tobacco legislation, and similarly to the teachers, in case of the break of regulations by the students, they need to react, report such incidents to the teachers, therefore, they are also responsible for shaping attitudes and social norms of the students. In small towns and villages, they are just as important as teachers because they are known by everybody and they are respected. Training is not only necessary in terms of gaining substantive knowledge on harmfulness of tobacco smoking, which may make teachers quit smoking themselves and pass this knowledge to students but also in terms of legislation and adherence to it.

As the study’s results have indicated, e.g., students who had good knowledge about harmful effects of smoking (OR = 0.54, 95% CI [0.43–0.69]); and had access to anti-smoking media (OR = 0.73, 95% CI [0.59–0.89]) were less likely to be susceptible to smoking [15]. Moreover, school policies banning smoking by teachers and other school personnel within and outside the school area are an important component of comprehensive adolescent smoking prevention programs. Poulsen et al. have found that the prevalence of smoking among adolescents was associated with a higher exposure to teachers smoking outdoors but not indoors [10]. It is possible that policies prohibiting outdoor smoking send a stronger “message” than those that only address indoor smoking. The latter may be perceived as “passive” adherence to legislation, while the former reflects a proactive position based on principle and on the acceptability and tolerance of smoking. Similarly, policies targeting staff, but not those targeting students, were associated with daily smoking, suggesting that policies that target students’ social environment, rather than those aimed directly at their behavior, may be more effective in reducing overall student smoking [22]. In general higher levels of perceived enforcement of anti-smoking policy at the school level were inversely associated with the prevalence of past-30-day smoking behaviors, independent of individual-level predictors [42]. Restrictions on smoking at home, more extensive bans on smoking in public places, and enforced bans on smoking at school may reduce teenage smoking [44]. The study by Nikaj and Chaloupka examinating the link between personnel and teacher smoking on school grounds, and student smoking in 62 low-income and middle-income countries has shown that smoking by personnel and teachers on school grounds is associated with higher smoking prevalence among all youths, and higher cigarette consumption among female smokers. In addition, they have found that smoking restrictions on staff are associated with reductions in average consumption among female students Their findings suggest that low-income and middle-income countries may reduce smoking among young people by banning smoking for teachers and school personnel on school grounds [43]. In the study conducted in China the likelihood of tobacco use was significantly higher among those having peers, teachers or mother who smoked [58].

An advantage of the current study is the fact that it is based on the standard GSPS methodology. Additional questions incorporated into the questionnaire made it possible to study the specific situation in Poland. In addition this study showed the situation in small cities usually poorly covered by surveillance, educational, promotional activities. It should be highlighted that with respect to public health activities and anti-tobacco programs, small towns and villages are straightaway in a worse situation, which results from the lower number of medical professionals and other resources (including human and financial) dedicated to prevention activities, worse access to medical units as well as institutions which are responsible for implementation of such measures. Also access to the Internet is still considerably reduced in comparison to the access in big cities, which makes it more difficult to use, e.g., social media for anti-tobacco initiatives. What is more, funds to be used for the purpose of prevention most frequently go to the centralized institutions that have their headquarters in big cities, which due to logistic and organizational issues, and also due to high population density and cost-effectiveness in big cities concentrate these type of actions in such centers. Inequalities concerning students access to prophylactic health care, with a worse situation of students of the village schools, vocational schools and special schools in the city are one of the symptoms of polarization and social differentiation in Poland. Those inequalities concern environments that have unequal development chances and are in danger of social exclusion. Inhabitants of outlying places use ‘services’, offers and ‘non-compulsory’ actions considerably less often (compulsory activities are, e.g., those associated with settling matters in an office or participation in so-called “parents’ evenings”, participation in outdoor parties or active participation in bicycle tours starting from school). In turn, in the outlying areas, activities related to health promotion are rare. The social networks in the rural areas are less extensive, while an extensive network of acquaintances, even if only superficial, usually presents various possibilities, brings about proposals for other activities and spending leisure time, participation in various types of events, undertakings, acquiring new ideas, knowledge, and information. The basic differences in the functioning of rural and urban children and adolescents concern the access to various places, public spaces, social contacts, experiences and also contact with young adults from whom ideas on other, more healthy lifestyles and activities may be taken. The gradually improving access to the Internet does not change the situation much. In the environment of rural school children there are hardly any people who could and would like to show them some interesting websites, expand their scope of interest in health and methods of its enhancement, and critically evaluate various sources of health information. The poor state of health education in rural schools in Poland is exacerbated by the reorganization of the network of educational facilities, especially elementary schools, justified by the rationalization of costs [35–38].

As the limitation of the study cross-sectional design need to be pointed however from a public health perspective, our data may be a sufficient reason to take some actions at local and national levels. The other limitation of the study is created by the fact that the school personnel participation was voluntary, therefore, it may be subject to selection bias. The participation rate was 83,1%. The findings are based on the self-reports from the staff who may under or over report their behavior and their knowledge or attitudes towards tobacco. The study does not include biochemical verification of the smoking status nor independent validation of school policies and enforcement of school tobacco control policies. Moreover, the study results reflect the situation in Piotrkowski district (which is socio-economically disadvantaged rural area) so their applicability to urban areas (or other regions) may be limited. Finally, a potential weakness of this study is that all school personnel may not be expected to have an equal role in providing tobacco control education. We can expect that a health teacher or a physical education teacher would spend more time discussing the health risks of smoking than a mathematics teacher. However, each teacher is a class tutor/form teacher responsible for the one class within the school and is responsible for upbringing and public health issues. Moreover there is 45 min/per week form period on which issues relating to young people upbringing and public health education need to be performed. This means that teachers should be prepared for this role. Looking at our results 22.5% of the teachers indicated that it is not their role to educate young people how to prevent the youth tobacco use. This can indicate that the teachers think that this is not their role to teach students about health related issues, while in fact this is their role (and they are not even aware of it).

## Conclusions

Our study indicates that there is an urgent need for taking actions aiming at increasing effectiveness of enforcing applicable tobacco control regulations in educational units. The necessity for systematic training dedicated to the youth to prevent their tobacco use, including accurate preparation of teachers, also needs to be highlighted.

## Additional files

Additional file 1: The dataset used for the analyzes within the study. (XLSX 705 kb)
Additional file 2: Table S1. Training and access to teaching materials according to the teachers smoking habits. (DOCX 15 kb)
