# Supplementary material for: Tobacco use patterns, knowledge, attitudes towards tobacco and availability of tobacco control training among school personnel from a rural area in Poland
Source: Tob Induc Dis. 2017 Jan 11;15:3. doi: 10.1186/s12971-016-0110-y (PMC5225631; doi:10.1186/s12971-016-0110-y)
Supplement: Additional file 2: Table S1. — Training and access to teaching materials according to the teachers smoking habits. (DOCX 15 kb) [file 12971_2016_110_MOESM2_ESM.docx]

Table S1. Training and access to teaching materials according to the teachers smoking habits

|  | Current smoker  N=221 | Ex-smoker  N=69 | Never smoker  N=754 | p-value |
| --- | --- | --- | --- | --- |
|  | N(%)(CI 95%) | N(%)(CI 95%) | N(%)(CI 95%) |  |
| **Information about health consequences of smoking given by professionals have impact on the youth’s decision about tobacco use (initiation or cessation of smoking)** | | | | |
| Yes | 108(48.9)  43.3-55.5 | 43(62.3)  50.9-73.7 | 422(56.0)  52.5-59.5 | 0.05^a^ |
| No | 37(16.7)  11.8-21.6 | 8(11.6)  4.0-19.2 | 103(13.7)  11.3-16.2 |  |
| I do not know | 76(34.4)  28.1-40.7 | 18(26.1)  15.7-36.5 | 229(30.4)  27.1-33.7 |  |
| **There is a need for training dedicated to the youth to prevent their tobacco use** | | | | |
| Yes | 187(84.6)  79.8-89.4 | 57(82.6)  73.6-91.6 | 871(89.0)  86.8-91.2 |  |
| No | 12(5.4)  2.4-8.4 | 5(7.2)  1.1-13.3 | 30(4.0)  2.6-5.4 |  |
| I do not know | 22(10.0)  6.0-14.0 | 7(10.1)  3.0-17.2 | 53(7.0)  5.2-8.8 |  |
| **Do the parents expect an in-school training for the youth to prevent their tobacco use?** | | | | |
| Yes | 123(55.7)  49.2-62.3 | 43(62.3)  50.9-73.7 | 430(57.0)  53.5-60.5 |  |
| No | 10(4.5)  1.8-7.2 | 7(10.1)  3.0-17.2 | 29(3.8)  2.4-5.2 |  |
| I do not know | 88(39.8)  33.3-46.2 | 19(27.5)  17.0-38.0 | 295(39.1)  35.6-42.6 |  |
| **Have knowledge necessary to educate how to prevent youth tobacco use** | | | | |
| Yes | 105(47.5)  40.9-54.1 | 30(43.5)  31.8-55.2 | 343(45.5)  42.0-49.1 |  |
| No | 69(31.2)  25.1-37.3 | 24(34.8)  23.6-46.0 | 238(31.6)  28.3-34.9 |  |
| It is not my role to conduct such activities | 47(21.3)  15.9-26.7 | 15(21.7)  12.0-31.4 | 173(22.9)  19.9-25.9 |  |
| **Source of knowledge about health consequences of smoking** | | | | |
| I do not have that information | 6(2.7)  0.6-4.8 | 3(4.3)  0.0-9.1 | 44(5.8)  4.1-7.5 |  |
| From TV | 118(53.4)  46.8-60.0 | 38(55.1)  43.4-66.8 | 405(53.7)  50.1-57.3 |  |
| From internet | 139(62.9)  56.5-69.3 | 51(73.9)  63.5-84.3 | 511(67.8)  64.5-71.1 |  |
| Special training (including the methods for antismoking counseling) | 42(19.0)  13.8-24.4 | 11(15.9)  7.3-24.5 | 191(25.3)  22.2-28.4 | 0.05^b^ |
| Leaflets | 155(70.1)  64.1-76.1 | 54(78.3)  68.6-88.0 | 571(75.7)  72.6-78.8 |  |
| Other | 19(8.6)  4.9-12.3 | 3(4.3)  0.0-9.1 | 19(2.5)  1.4-3.6 | 0.04^b^ |
| **Who have organized the training how to prevent the youth tobacco use** | | | | |
| I have not participated in such training | 150(67.9)  61.7-74.1 | 48(69.6)  58.8-80.5 | 475(63.0)  59.6-66.5 |  |
| It was organized by school | 28(12.7)  8.3-17.1 | 8(11.6)  4.0-19.2 | 119(15.8)  13.2-18.4 |  |
| By State Board of Education | 6(2.7)  0.6-4.8 | 1(1.4)  0.0-4.2 | 36(4.8)  3.3-6.3 |  |
| By Self-Government Authorities | 24(10.9)  6.8-15.0 | 7(10.1)  3.0-17.2 | 89(11.8)  9.5-14.1 |  |
| It was the part of obligatory trading for teachers | 13(5.9)  2.8-9.0 | 5(7.2)  1.1-13.3 | 35(4.6)  3.1-6.1 |  |
| **Taught the youth how to avoid tobacco use** | | | | |
| Yes during regular classes | 197(89.1)  85.0-93.2 | 60(86.9)  78.9-94.9 | 651(86.3)  83.8-88.8 |  |
| Yes but additionally to regular classes | 33(14.9)  10.2-19.6 | 14(20.3)  10.8-29.8 | 193(25.6)  22.5-28.7 | 0.001^b^ |
| No | 14(6.3)  3.1-9.5 | 4(5.8)  0.3-11.3 | 47(6.2)  4.5-7.9 |  |
| **Non-classroom activities to teach about tobacco use and its prevention** | | | | |
| Yes | 189(85.5)  80.9-90.1 | 55(79.7)  70.2-89.2 | 607(80.5)  77.7-83.3 |  |
| No | 32(14.5)  9.9-19.1 | 14(20.3)  10.8-29.8 | 147(19.5)  16.7-22.3 |  |

a Current smokers vs. ex-smokers

b Current smokers vs. never smokers
